# Behavioral characteristics of dopamine D_5_ receptor knockout mice

**DOI:** 10.1038/s41598-022-10013-5

**Published:** 2022-04-10

**Authors:** Hitomi Sasamori, Toshiaki Asakura, Chiaki Sugiura, Youcef Bouchekioua, Naoya Nishitani, Masaaki Sato, Takayuki Yoshida, Miwako Yamasaki, Akira Terao, Masahiko Watanabe, Yu Ohmura, Mitsuhiro Yoshioka

**Affiliations:** 1 Department of Neuropharmacology, Hokkaido University Faculty of Medicine, Sapporo, Japan; 2 Hokkaido University School of Medicine, Sapporo, Japan; 3 Laboratory of Molecular Pharmacology, Institute of Medical, Pharmaceutical and Health Sciences, Kanazawa University, Kanazawa, Japan; 4 Department of Neurophysiology, Graduate School of Biomedical and Health Sciences, Hiroshima University, Hiroshima, Japan; 5 Department of Anatomy, Hokkaido University Faculty of Medicine, Sapporo, Japan; 6 Department of Biology, School of Biological Sciences, Tokai University, Sapporo, Japan

**Keywords:** Cognitive neuroscience, Learning and memory, Genetics of the nervous system

## Abstract

Major psychiatric disorders such as attention-deficit/hyperactivity disorder and schizophrenia are often accompanied by elevated impulsivity. However, anti-impulsive drug treatments are still limited. To explore a novel molecular target, we examined the role of dopamine D_5_ receptors in impulse control using mice that completely lack D_5_ receptors (D5KO mice). We also measured spontaneous activity and learning/memory ability because these deficits could confound the assessment of impulsivity. We found small but significant effects of D_5_ receptor knockout on home cage activity only at specific times of the day. In addition, an analysis using the q-learning model revealed that D5KO mice displayed lower behavioral adjustment after impulsive actions. However, our results also showed that baseline impulsive actions and the effects of an anti-impulsive drug in D5KO mice were comparable to those in wild-type littermates. Moreover, unlike previous studies that used other D_5_ receptor-deficient mouse lines, we did not observe reductions in locomotor activity, working memory deficits, or severe learning deficits in our line of D5KO mice. These findings demonstrate that D_5_ receptors are dispensable for impulse control. Our results also indicate that time series analysis and detailed analysis of the learning process are necessary to clarify the behavioral functions of D_5_ receptors.

## Introduction

Various psychiatric disorders, such as attention-deficit/hyperactivity disorder (ADHD), schizophrenia, substance use disorder, bipolar disorder, and borderline personality disorder, have been associated with increased impulsivity^1^. Clinically available anti-impulsive drugs vary from country to country, but at present, the major ones include amphetamine, methylphenidate, and lisdexamfetamine as psychostimulants, and atomoxetine, guanfacine, and clonidine as adrenaline-related drugs. However, psychostimulants pose a risk of abuse and dependence, and adrenaline-related drugs are often difficult to recommend due to their cardiovascular and autonomic side effects. For example, atomoxetine, a noradrenaline reuptake inhibitor, might not be appropriate in some patients since it can exacerbate hypertension, which is often comorbid with ADHD, bipolar disorder, and borderline personality disorder^2–4^. Although guanfacine and clonidine are hypotensive agents, their sedative side effects could interfere with work or study. Therefore, further development of novel anti-impulsive drugs is required.

To this end, we examined whether dopamine D_5_ receptors play an important role in the control of impulsivity. We have previously demonstrated that dopamine D_1_-like receptors in the ventral part of the medial prefrontal cortex play a critical role in the anti-impulsive effects of milnacipran, duloxetine, and atomoxetine^5,6^. There are two types of dopamine D_1_-like receptors: D_1_ receptors and D_5_ receptors. Dopamine D_1_ receptors are densely expressed in the nucleus accumbens, where impulsivity is enhanced by increased extracellular dopamine levels^7,8^, while dopamine D_5_ receptors are sparsely expressed in the region^9^. In comparison, in the medial prefrontal cortex, where impulsivity is inhibited by increased extracellular dopamine levels^10,11^, both dopamine D_1_ and dopamine D_5_ receptors are expressed^9^. Furthermore, dopamine D_5_ receptors have a tenfold higher affinity for dopamine than dopamine D_1_ receptors^12^. Thus, we hypothesized that the anti-impulsive effects of the above drugs might be exerted via stimulation of dopamine D_5_ receptors.

The development of a selective dopamine D_5_ receptor agonist might resolve problems encountered with the current stable of anti-impulsive drugs by enabling us to selectively manipulate the medial prefrontal cortex without affecting the nucleus accumbens. To our knowledge, however, there are so far no drugs that clearly distinguish between dopamine D_1_ and D_5_ receptors. Given that psychostimulants primarily facilitate addiction through the modulation of the nucleus accumbens^13^, a selective dopamine D_5_ receptor agonist might not induce this process, unlike psychostimulant-based anti-impulsive drugs. Furthermore, a selective agonist for dopamine D_5_ receptors would not likely exacerbate hypertension since dopamine D_5_ receptor knockout (D5KO) mice are hypertensive^14^. Spontaneous motor activity in D5KO mice is generally normal or reduced, implying that a selective dopamine D_5_ receptor agonist will not induce sedation. However, in the absence of selective D5 receptor agonists, examining dopamine D5KO mice is a reasonable way to determine whether D_5_ receptors could be a promising target for anti-impulsive drugs.

In the present study, we used an alternative line of D5KO mice^15^ instead of traditional D5KO mice^14^ because of three reasons. First, the traditional D5KO mice could express truncated transcripts that might alter the expression of related genes^16,17^, while the alternative line of D5KO mice would not express them because the entire dopamine D_5_ receptor gene region, including the promoter region, is removed. Second, previous studies have shown that different lines of transgenic mice or different background strains of transgenic mice could alter baseline behavioral phenotype^18,19^. To clarify the role of a molecule in brain functions, we are better off testing not only a specific line or background strain but also another line or background strain. Third, some studies have reported lower spontaneous motor activity and deficits of learning and working memory in traditional D5KO mice^20–22^. These phenotypes make it difficult for researchers to assess impulsivity because most tasks evaluating impulsivity assume a certain level of spontaneous activity and learning/memory ability. We speculated that these phenotypes are due to the above reasons, but not due to the lack of D_5_ receptors.

In this study, using an alternative line of D5KO mice, we conducted quantitative PCR to confirm that dopamine D_5_ receptors were not transcribed as expected and whether compensatory changes in dopamine D_1_ receptors did not occur, (2) measured locomotor activity in two different environments: a novel environment and a familiar environment, (3) conducted a Y-maze test to assess working memory, and (4) employed the 3-choice serial reaction time task (3-CSRTT)^11,23^ to assess learning ability and impulsivity. To evaluate possible learning deficits or bias, we modeled the learning process within the 3-CSRTT using a q-learning model.

## Results

### RNA analysis

**Figure 1:**
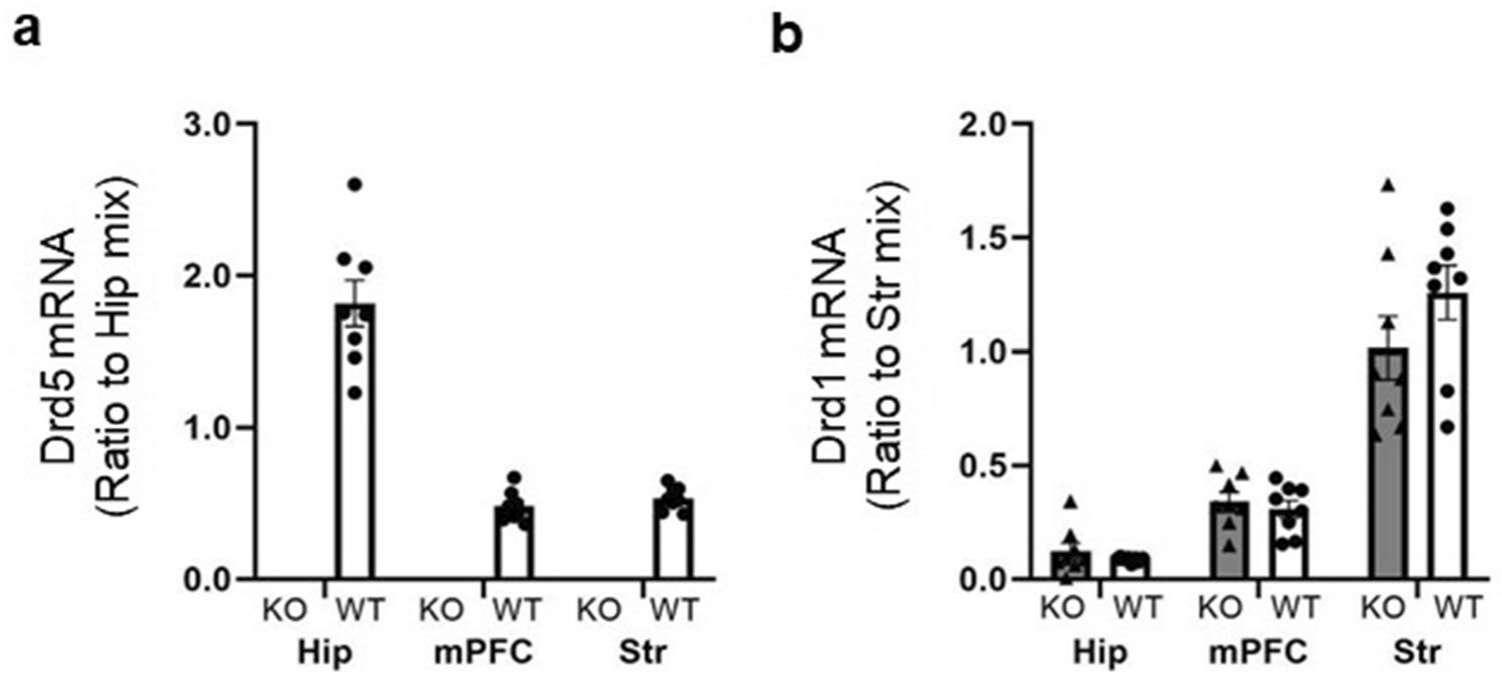
Effects of genotype on *Drd5* and *Drd1* gene expression. (**a**) *Drd5* gene relative expression levels in D5KO (KO, dark bars) mice and wildtype (WT, white bars) littermates. (**b**) *Drd1* gene relative expression levels in D5KO (KO, dark bars) mice and WT (white bars) littermates. Mix means a mixture of KO and wildtype samples. Hip: hippocampus, mPFC: medial prefrontal cortex, Str: striatum. The data are presented as the means ± SEM.

To confirm that the dopamine D_5_ receptor gene is not transcribed and that a compensatory overexpression of D_1_ receptors does not occur, we conducted quantitative PCR tests. As expected, the *Drd5* gene expression levels were below the detection limit in the D5KO mice (Fig. 1a). Moreover, the *Drd1* gene expression levels were not increased in the D5KO mice compared to wildtype littermates in the hippocampus (*t*_14_ = 0.96, *p* = 0.35), medial prefrontal cortex (mPFC) (*t*_14_ = 0.61, *p* = 0.55), and striatum (*t*_14_ = -1.34, *p* = 0.20) (Fig. 1b).

### Home cage activity

**Figure 2:**
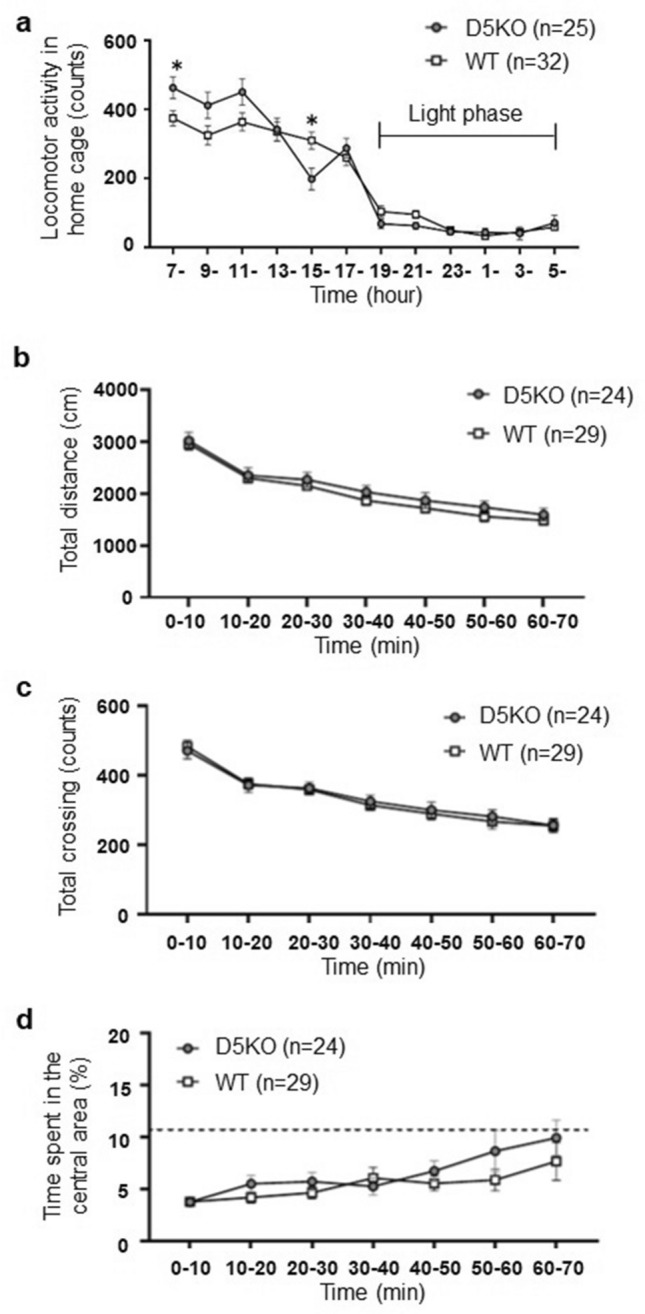
Effects of genotype on 24 h locomotor activity in home cages and on parameters in the open field test. (**a**) Home cage locomotor activity of D5KO mice and wildtype littermates every 2 h. *p < 0.05. (**b**) The total distance traveled in the open field test was divided into seven time phases (10 min bins). (**c**) The number of total crossings (crossings of the lines made by the division of the field [45 × 45 cm] into 7.5 cm × 7.5 cm squares) in the open field test was divided into seven time phases (10 min bins). (**d**) The percentage of time spent in the central area, a measure of decreased anxiety-like behavior in the open field test, was divided into seven time phases (10 min bins). The filled circles indicate D5KO mice and white squares indicate WT littermates. The data are presented as the means ± SEM.

To measure locomotor activity in a familiar environment, we measured home cage activity for 24 h. We performed a three-factor ANOVA on the changes in locomotor activity every two hours in their home cages (Fig. 2a). There was a main effect of time (*F*_5.39, 285.43_ = 115.28, *p* < 0.001, with Greenhouse–Geisser correction). There was a significant interaction between time and genotype (*F*_1, 53_ = 3.34, *p* < 0.001, with Greenhouse–Geisser correction). Other main effects and interactions were not detected (Table S1).

Simple main effects analyses for each time point revealed that D5KO mice were significantly more active than wildtype littermates between 7:00 and 9:00 (*F*_1, 53_ = 4.63, *p* = 0.036) (Fig. 2a). D5KO mice were also significantly less active than wildtype littermates between 15:00–17:00 (*F*_1, 53_ = 6.88, *p* = 0.011) (Fig. 2a). No differences by genotype at other times were detected (*F*s_1, 53_ < 3.19, *p* > 0.07).

### Open field test

To measure locomotor activity in a novel environment, we conducted open field tests for 70 min. Three factor ANOVA revealed that the distance traveled over the testing period significantly decreased over time (*F*_3.45, 169_ = 119.1, *p* < 0.0001, with Geisser-Greenhouse correction). However, we found no significant main effects or interactions in other factors (Fig. 2b, Table S2).

A three-factor ANOVA for the number of crossings revealed a significant main effect of time (*F*_6, 294_ = 93.96, *p* < 0.0001). However, we found no significant main effects or interactions in other factors (Fig. 2c, Table S2).

A three-factor ANOVA for the percentage of time spent in the central area, which is a measure of decreased anxiety-like behavior, revealed a significant main effect of time (*F*_2.99, 146.5_ = 6.86, *p* = 0.0002, with Geisser-Greenhouse correction). However, we did not find other main effects or any interactions (Fig. 2d, Table S2).

### Y maze test

**Figure 3:**
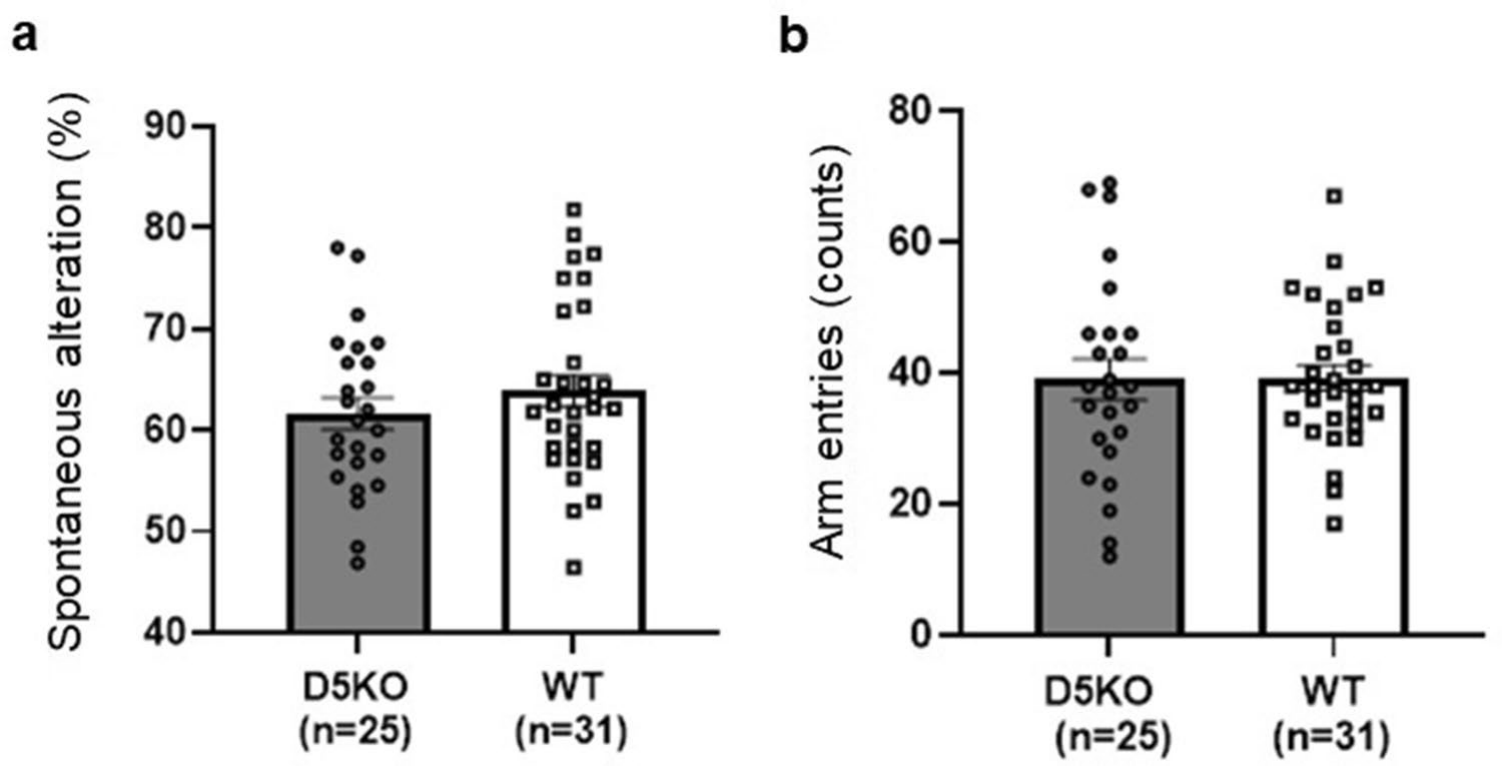
The effects of dopamine D_5_ receptor KO on the parameters in the Y maze test. (**a**) The percentage of spontaneous alternation, a measure of working memory, in D5KO mice and their WT littermates. (**b**) The total number of arm entries, a measure of locomotor activity, of D5KO mice and their WT littermates. The data are presented as the means ± SEM.

To assess working memory in mice, we conducted the Y maze test. Two factor ANOVA for the percentage of spontaneous alternation, a measure of working memory, did not reveal any main effects or interaction (Fig. 3a, Table S3). Two factor ANOVA for the total number of arm entries, a measure of locomotor activity, did not reveal any main effects or interaction (Fig. 3b, Table S3).

### Assessment of learning ability with a q-learning model

To determine whether D5KO mice display learning deficits or bias, we modeled the learning process of 3-CSRTT. We assume experience and non-reward distributions to represent premature behaviors of mice. Experience distribution represents a memory state of all trials and is updated by the q-learning process whatever a previous result is, while non-reward distribution represents a memory state of premature responses. Table 1 shows the estimated parameters of a q-learning model, and Table S4 has descriptions of each parameter. In this model, key parameters are learning rates, representing learning ability, and an inverse temperature, representing confidence in their own choice. The baseline effect of learning rate for the experience distribution, ${\alpha }_{X,0},$ was 0.04296 while that for the non-reward distribution, ${\alpha }_{Y,0}$, was 0.08555. For the experience distribution, only male effects were significant according to the 95% Highest Density Interval (HDI). Also, for the non-reward distribution, both D5KO and male effects were negative and significant, based on 95% HDI. This result indicated that D5KO mice have a learning deficit for premature results, not for non-premature results. As for the inverse temperature, baseline effect, ${\beta }_{0}$, took a value of 130.603, and both D5KO and male effects were not significant.

**Table 1:** Estimated parameters of the q-learning model.

	Mean	95% HDI^a^
${\alpha }_{X,0}$	0.04296	(0.03829, 0.04784)
${\alpha }_{X, D5KO}$	− 0.00147	(− 0.00379, 0.00058)
${\alpha }_{X,male}$	− 0.00612	(− 0.00895, − 0.00372)
${\alpha }_{Y,0}$	0.08555	(0.07465, 0.09726)
${\alpha }_{Y, D5KO}$	− 0.00517	(− 0.00782, − 0.00260)
${\alpha }_{Y,male}$	− 0.01394	(− 0.01726, − 0.01087)
${\beta }_{0}$	130.603	(117.447, 144.407)
${\beta }_{D5KO}$	9.925	(− 2.731, 22.702)
${\beta }_{male}$	− 5.961	(− 20.987, 8.380)
${\alpha }_{M}$	0.03755	(0.03097, 0.04463)
${\sigma }_{M}^{2}$	617.027	(526.455, 718.198)
${\sigma }_{r}^{2}$	433.462	(373.520, 502.798)
${\sigma }_{X,0}^{2}$	15.816	(12.792, 19.261)
${\sigma }_{Y,0}^{2}$	52.144	(43.677, 62.984)

It is noted that ${\alpha }_{X, D5KO}, {\alpha }_{X,male}, {\alpha }_{Y, D5KO},$ and ${\alpha }_{Y,male}$ were sampled from real numbers, while the other parameters were sampled from positive real numbers.

^a^HDI represents the highest density interval.

Although ${\alpha }_{X, male}$, ${\alpha }_{Y, D5KO,}$ and ${\alpha }_{Y, male}$ were not zero for these 95% HDI, the degree of effects of these parameters remains unclear. To quantify contributions of these parameters, we simulated the q-learning process with estimated parameters. Table S5 shows proportions of premature responses for each session for trial and simulation data. For the simulation data, overall, the start timing of the proportions was consistently lower than those for trial data. The proportions of premature responses among each session fluctuated for real and simulated results. If we focus on the values at session 10, the minimum values were observed in D5KO male mice, whereas the maximum value was observed in wildtype female mice. Simulated results with individually estimated parameters using the q-learning model over trials indicated the model could potentially capture behaviors of trial results in the 3-CSRTT (see Supplementary Fig. 1).

**Figure 4:**
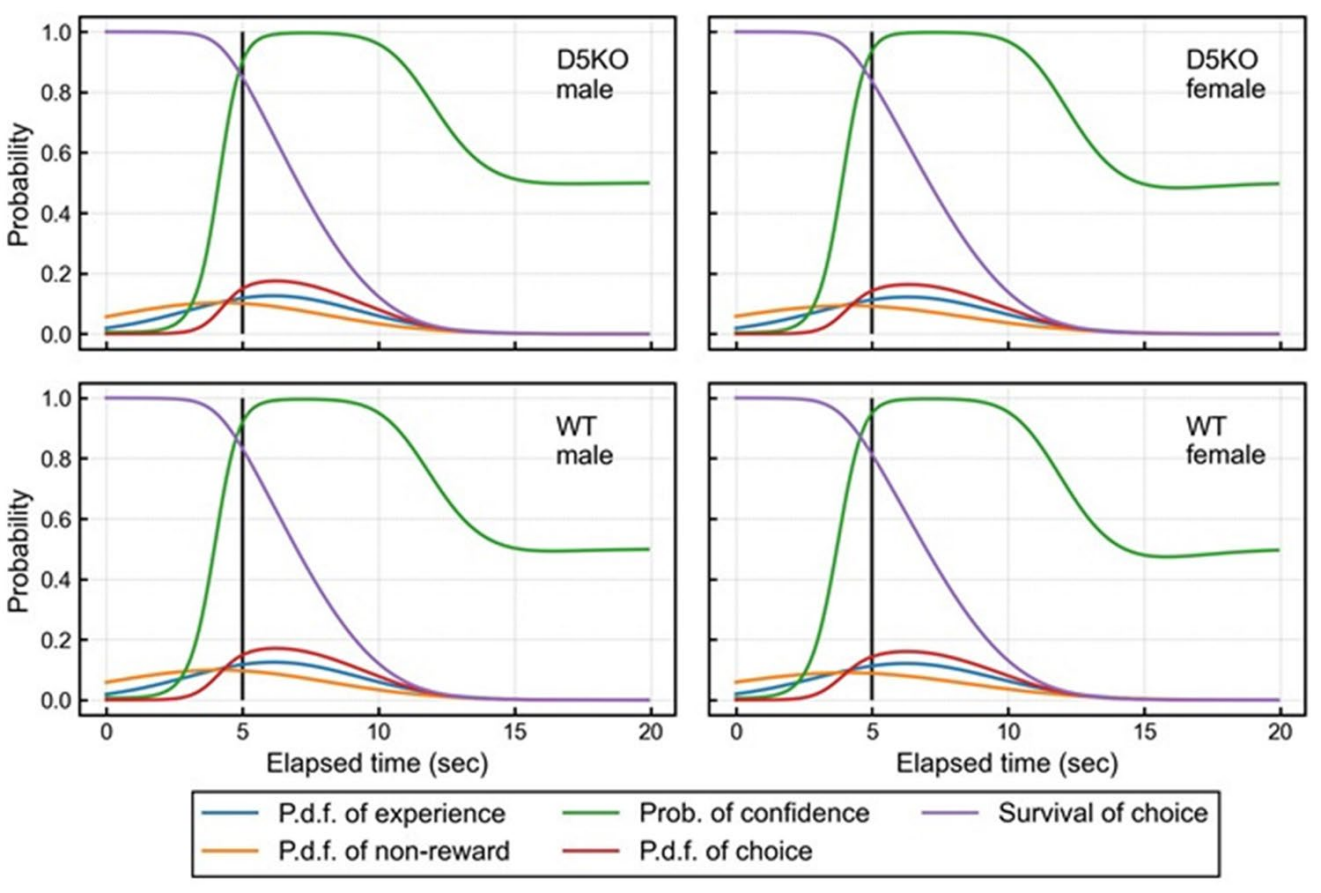
Averaged function values for the q-learning model at the end of the simulation with estimated parameters. Each simulation was run for 900 steps and 100 simulations were performed. Figure contains averaged values for these simulations at each elapsed time (s). The black line represents the flash timing of the trial. Each line represents the following: Probability density function (p.d.f.) of experience (blue); the p.d.f. of experience distribution. P.d.f. of non-reward (orange); the p.d.f. of non-reward distribution. Prob. of confidence (green); the probability of confidence at each elapsed time. P.d.f. of choice (red); the p.d.f. of choice distribution. Survival of choice (purple): the survival function of the choice distribution.

Averaged values for functions used in the q-learning model at the end of the simulation with estimated parameters are shown in Fig. 4. Values at each elapsed time were averaged values calculated from 100 simulation results. Overall, there were no major differences in the shape of the functions. For the probability of confidence, the rise of the distribution around 5 s was steeper in D5KO mice than in wild type mice, reflecting the higher value of inverse temperature, although the D5KO effect for an inverse temperature is not significant.

### Effects of acute duloxetine administration on impulsive action

**Figure 5:**
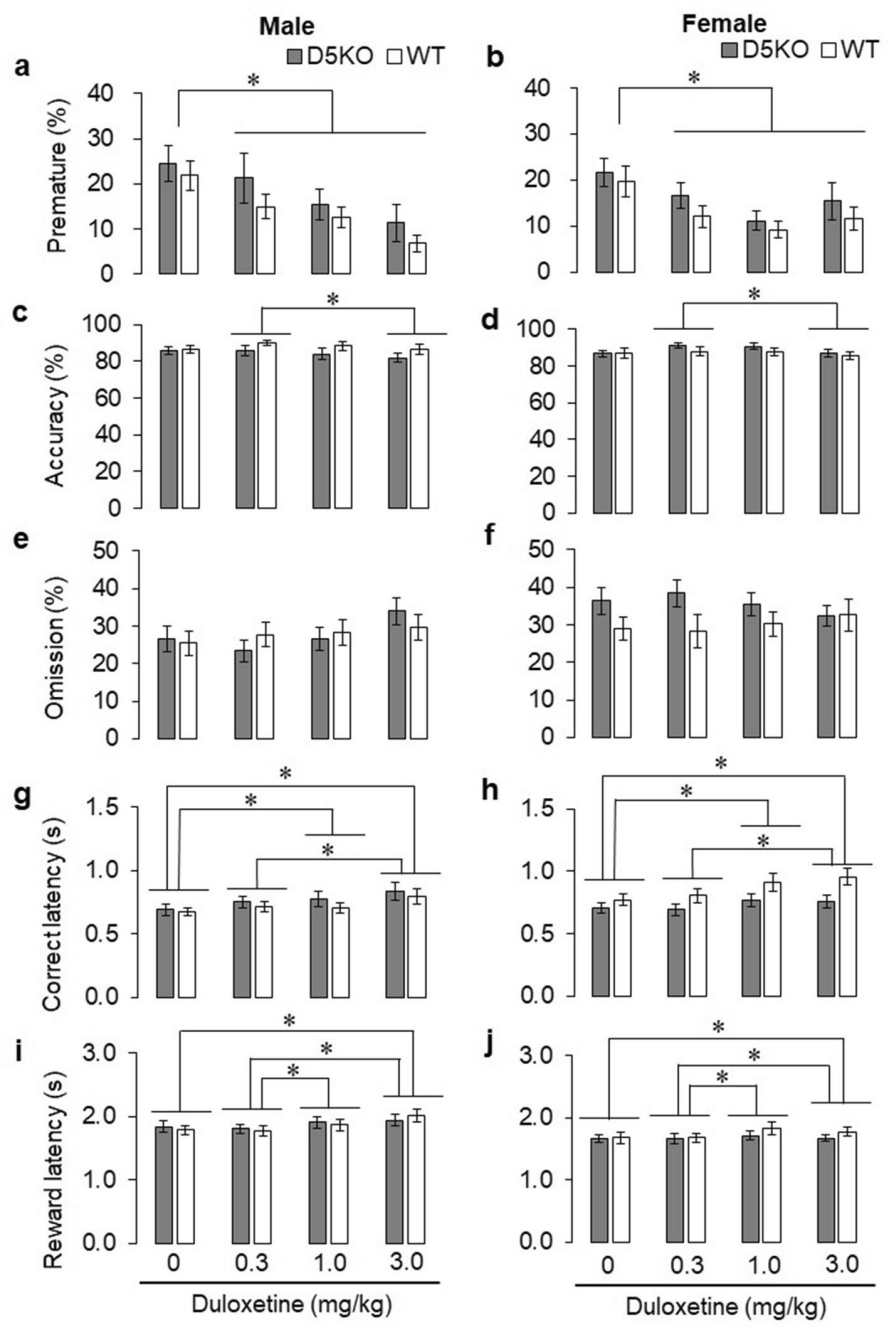
Effects of acute duloxetine administration on impulsive action. (**a**) The percentage of premature responses, a measure of impulsive action, of male D5KO mice and their WT littermates. (**b**) The percentage of premature responses, a measure of impulsive action, of female D5KO mice and their WT littermates. (**c**) Accuracy, the percentage of correct responses of male D5KO mice and their WT littermates. (**d**) Accuracy, the percentage of correct responses of female D5KO mice and their WT littermates. (**e**) The percentage of omissions of male D5KO mice and their WT littermates. (**f**) The percentage of omissions of female D5KO mice and their WT littermates. (**g**) The mean correct response latency (s) of male D5KO mice and their WT littermates. (**h**) The mean correct response latency (s) of female D5KO mice and their WT littermates. (**i**) The mean reward response latency (s) of male D5KO mice and their WT littermates. (**j**) The mean reward response latency (s) of female D5KO mice and their WT littermates. The black bars indicate D5KO mice and white bars indicate WT littermates. The data are presented as the means ± SEM. *p < 0.05.

To assess the effects of D5KO on impulsive action and anti-impulsive effects of duloxetine, we administered duloxetine and conducted the 3-CSRTT. Duloxetine administration reduced the percentage of premature responses in a dose-dependent manner (Fig. 5a,b). Three factor repeated measures ANOVA revealed a significant dose effect on the percentage of premature responses (Fig. 5a,b) (*F*_2.51, 140.26_ = 14.06, *p* < 0.001, with Greenhouse–Geisser correction). A multiple comparison with Bonferroni's correction revealed that the 0.3 mg/kg, 1.0 mg/kg, and 3.0 mg/kg dose of duloxetine significantly decreased the percentage of premature responses compared to vehicle administration. However, we did not find any other main effects or interactions (Table S6).

Furthermore, other parameters were also affected by the administration of duloxetine. Three factor repeated measures ANOVA revealed a significant dose effect on accuracy (Fig. 5c,d) (*F*_2.56_, _143.28_ = 3.93, *p* = 0.014, with Greenhouse–Geisser correction), correct response latency (Fig. 5g,h) (*F*_2.40_, _134.19_ = 10.66, *p* < 0.001, with Greenhouse–Geisser correction), and reward latency (Fig. 5i,j) (*F*_3, 168_ = 6.74, *p* < 0.001), but not on omission (Fig. 5e,f). Multiple comparisons with Bonferroni’s correction revealed that the 3.0 mg/kg dose of duloxetine decreased accuracy compared to the 0.3 mg/kg dose (Fig. 5c,d), while the 1.0 mg/kg dose of duloxetine prolonged correct response latency compared to vehicle, the 3.0 mg/kg dose of duloxetine prolonged correct response latency compared to vehicle and the 0.3 mg/kg dose (Fig. 5g,h), the 3.0 mg/kg dose of duloxetine administration significantly prolonged reward latency compared to vehicle and 0.3 mg/kg duloxetine, and the 1 mg/kg duloxetine administration significantly prolonged the reward latency compared to 0.3 mg/kg (Fig. 5i,j). Moreover, a main effect of sex was detected on mean reward response latency (*F*_1, 56_ = 5.210, *p* = 0.026). However, we did not find any other main effects or interactions (Table S6).

## Discussion

Although minor differences were found, no major differences were observed in any behavioral parameters between wildtype and D5KO mice. Small but significant effects of D5KO were observed in home cage activity only at specific times of day. In addition, D5KO mice displayed lower behavioral adjustments after premature responses in the 3-CSRTT. We did not observe a reduction in locomotor activity in a novel environment or working memory deficits in D5KO mice, inconsistent with some previous studies. No significant effects of D5KO on impulsive action were observed, suggesting that our hypothesis that D_5_ receptors play an essential role in impulse control is incorrect. We discuss possible interpretations for each result below.

We replicated a previous KO study that indicated no mRNA expression of D_5_ receptors using Northern blotting^15^. We used a different method, quantitative RT-PCR (Fig. 1a) and reached the same conclusion: our D_5_ receptor KO mice do not express D_5_ receptor at all. Another concern of D_5_ receptor KO mice is compensatory effects. For decades, studies from transgenic and gene knockout mice have contributed to the delineation of the functional role of many kinds of proteins. However, recent evidence has demonstrated that the interpretation of these studies may be complicated by compensatory changes in animals because gene mutations truncating the encoded protein could affect the expression of related genes^16,17^. Our RT-qPCR results (Fig. 1b) indicated that we could exclude the possibility of a compensatory increase of dopamine D_1_ receptors, which are involved in impulse control. However, we cannot deny the other numerous possibilities that expression of other genes^24^ or functional pathways^25,26^ was altered in D_5_ receptor KO mice. In future studies, an AAV-mediated knockdown or knockout of the D5 receptor in the PFC would be required to ensure no compensatory changes because testing above numerous possibilities is impractical.

We found that dopamine D_5_ receptor KO mice exhibited higher locomotor activity at the beginning of the dark period (7:00–9:00) but lower locomotor activity at the end of the dark period (15:00–17:00) in their home cage, a familiar environment (Fig. 2a). Previous studies have shown that dopamine D_5_ receptor KO mice display lower locomotor activity than wildtype mice^21,22^, while others did not detect any difference in locomotor activity^15,20^. The present study might explain the inconsistent results from previous studies on locomotor activity in dopamine D_5_ receptor KO mice. In the previous studies, an open field test lasting 60 to 150 min has been used to measure locomotor activity. However, since the locomotor activity of dopamine D_5_ receptor KO mice changed significantly between the first and second halves of the dark period, the results may vary depending on the time of day the test was conducted. Although speculative, previous studies that showed lower locomotor activity in dopamine D_5_ receptor KO mice might have been conducted in the latter half of the dark period.

In the open field test in the present study, we did not detect any difference in locomotor activity in the novel environment between dopamine D5KO mice and their wild type littermates (Fig. 2b,c). This might be due to the fact that the time of measurement was not kept constant. Alternatively, the results in the home cage described above might be limited to a familiar environment. Because further studies examining locomotor activity in the open field at specific times will be required to address this issue, we suspend our conclusion. In addition, there was no difference in the time spent in the central compartment (%), a measure of reduced anxiety-like behavior, in dopamine D_5_ receptor KO mice compared to their wildtype littermates (Fig. 2d). Therefore, our findings indicate that dopamine D_5_ receptors may not relate to anxiety-like behavior, consistent with previous studies^21,22^.

In the Y maze test, there was no difference in working memory in dopamine D_5_ receptor KO mice compared to their wildtype littermates (Fig. 3). However, in previous studies, dopamine D_5_ receptor KO mice tended to exhibit lower working memory^20–22^. There are at least two possible explanations for this discrepancy. First, we used an alternative line of D_5_ receptor KO mice in this study^15^, while the previous studies that detected working memory deficits had used traditional dopamine D_5_ receptor KO mice^14^. As discussed earlier, the traditional mice could alter the expression of related genes^16,17^. Thus, working memory deficits observed in these studies might be due to the secondary effects. The second possibility is the difference in the working memory measurement task employed. A previous study demonstrating working memory deficit in D_5_ receptor KO mice used a baited T-maze test^20^. In the present study, we used the Y maze test as a simple test that does not require training. In this test, behavioral variability would be relatively large because we do not provide a clear motivation such as a food reward. Therefore, the Y maze test might not be able to detect minute differences, though the Y maze test in our laboratory can detect working memory deficits by pharmacological manipulation^27^. Therefore, we conclude that the role of dopamine D_5_ receptors in working memory is limited.

Because our dopamine D5KO mice showed almost normal motor functions and working memory, we conducted the 3-CSRTT to assess impulsive actions. The q-learning analysis revealed that small deficits of learning were observed in D5KO mice (Table 1). In other words, D5KO mice, especially male mice, exhibit an inferior ability to learn from their mistakes and fine-tune their behavior. However, these small differences did not significantly affect behavioral parameters in the 3-CSRTT (Fig. 4). It should be also noted that the variability in the results of each individual mouse and each session is quite high. At a minimum, we raise the possibility that the q-learning model is useful for the analysis of learning processed in the 3-CSRTT, and the detailed time series analysis could provide a clue to clarify the function of D5 receptors.

We replicated the dose-dependent anti-impulsive effects of duloxetine previously found in male rats^5^ using male and female mice. However, the anti-impulsive effects of duloxetine were detected not only in the wild type littermates but also in dopamine D_5_ receptor KO mice. That is, D_5_ receptor KO failed to prevent the anti-impulsive effects of duloxetine, indicating that our original hypothesis was incorrect. Moreover, the baseline of impulsive action (following 0 mg of duloxetine) was almost the same between D_5_ receptor KO mice and wild type littermates. Based on these results, we suggest that dopamine D_5_ receptors do not play an important role in impulsivity. It should be noted that other parameters were also affected by duloxetine. Accuracy, a measure of attentional function, was decreased when 3 mg/kg duloxetine was injected in both genotype and both sexes. In addition, 1 mg/kg and 3 mg/kg duloxetine administration prolonged the mean correct latency and reward latency in both genotype and both sexes. These measures represent motivation and motor function. The percentage of omissions, which represents attentional function and motivation, was not affected by duloxetine. Therefore, the prolonged latencies would reflect a decrease in motor function, indicating that higher doses of duloxetine would be inappropriate in the evaluation of anti-impulsive effects. However, we still conclude that dopamine D_5_ receptors have a negligible role in impulse control because these side effects were equally observed in either genotype and the anti-impulsive effects of a low or moderate dose of duloxetine did not disappear in D5KO mice.

In light of these results, how should we interpret previous studies^5,6^ indicating that drugs suppress impulsivity by stimulating dopamine D_1_-like receptors in the mPFC? There are at least two possibilities. First, dopamine D_1_ receptors may be more involved in impulsivity suppression, since the involvement of D_5_ receptors has been ruled out. However, since dopamine D_1_ receptors are also densely expressed in the nucleus accumbens, where impulsivity is enhanced by their stimulation^7,8^, they will not be an appropriate molecular target for anti-impulsive drugs. The second possibility is that previous studies have largely examined nonselective effects of dopamine D_1_-like receptor antagonists, where SCH23390 is frequently used, although its selectivity for D_1_-like receptors is not high enough to completely exclude effects on other receptors and channels^28^. In either case, the development of a selective dopamine D_5_ receptor agonist would not resolve the current problems encountered in current anti-impulsive drugs. Interestingly, a recent study showed that striatal dopamine D_5_ receptors are involved in the pathophysiology of levodopa-induced dyskinesia^29^. Therefore, dopamine D_5_ receptors might play a role in pathological but not physiological situations.

## Materials and methods

### Animals

Adult male and female D5KO mice^15^ or wildtype littermates (8–28 weeks old) were used. The B6.129-Drd5 < tm1Mok > mouse strain (RBRC01084) was provided by RIKEN BRC through the National Bio-Resource Project of the MEXT, Japan. In the D5KO mice used in this study, the entire dopamine D_5_ receptor gene region was removed and replaced with a neomycin resistance gene. These mice were backcrossed to the C57BL/6N strain for more than 13 generations. C57BL/6N mice were supplied from Nippon SLC Co. Ltd (Hamamatsu, Japan). Animals were group-housed before starting behavioral experiments at 25 °C ± 2 °C and relative humidity of 40%–50%. Food and water were provided ad libitum except for the mice undergoing the 3-choice serial reaction time task. The lights of the animal rooms were turned on from 19:00 to 07:00. All tests were performed during the dark period except for the home cage activity test. All procedures followed the guidelines for the Care and Use of Laboratory Animals from the Animal Research Committee of Hokkaido University and were approved by the Animal Research Committee of Hokkaido University (approval no. 18-0070). We conducted all experiments in compliance with the Animal Research: Reporting of In Vivo Experiments (ARRIVE) guidelines. Mice received one or several behavioral tests as summarized in Table 2. A few mice that experienced the 3-choice serial reaction time task were excluded from assessment of learning ability because a programming error affected the premature response latency data.

**Table 2:** Grouping of mice.

	Wildtype littermates		D_5_ receptor KO mice	
	Male	Female	Male	Female
RNA analysis	4	4	4	4
Home cage activity	13	19	13	12
Open field test	12	17	12	12
Y maze test	12	19	13	12
Assessment of learning process in 3-choice serial reaction time task	14	11	11	10
3-choice serial reaction time task Effects of acute duloxetine injection on impulsive action	16	12	16	16

The left column indicates the tests that mice on the right received. The numbers in each group are slightly different because we used littermates in each experiment.

### Drugs

Duloxetine hydrochloride (Tokyo Chemical Industry Co., Ltd., Tokyo, Japan) was dissolved in saline and administered intraperitoneally at a volume of 10 mL/kg. Doses reported here are based on the molecular weight of the salt.

### RNA analysis

Mice were deeply anesthetized with urethane (2 g/kg) intraperitoneally and sacrificed by decapitation. Brain tissue including hippocampus, medial prefrontal cortex (mPFC), and striatum (Str) were dissected on ice. Each sample was weighed, placed in a tube, immediately frozen in liquid nitrogen and kept frozen at − 80 °C until analysis. Total RNA was extracted from tissue using NucleoSpin RNA reagent (Takara Bio, Shiga, Japan). The mRNA expression levels of *Drd1* and *Drd5* were quantified by reverse-transcription quantitative PCR (RT-qPCR) using the respective cDNA fragment as a standard and were normalized to mouse *Gapdh* mRNA levels. Briefly, 5 μg of total RNA were reverse transcribed using ReverTra Ace® qPCR RT Master Mix with gDNA Remover (Toyobo, Osaka, Japan). Real-time quantitative PCR was performed on a fluorescence thermal cycler Step One™ Real-time PCR System (Thermo Fisher Scientific, Waltham, MA, USA) by using TaqMan^®^ Fast Advanced Master Mix + probe set (Thermo Fisher Scientific). The PCR conditions were 50 °C for 2 min, 95℃ for 20 s, followed by 40 cycles of 95 °C for 1 s, and 60 °C for 20 s. Primer sequences for *Drd1* (Thermo Fisher Scientific, Mm01353211_m1) and *Drd5* (Thermo Fisher Scientific, Mm00658653_s1) were chosen based on a previous study^30^. *Gapdh* was used as a control (Thermo Fisher Scientific, Mm99999915_g1). The results were analyzed using the StepOne Software ver.2.3 (Thermo Fisher Scientific).

### Home cage activity

Animals were individually housed in a Plexiglas cage (18 cm × 26 cm × 12 cm) for at least 1 week before this test. Spontaneous movements were measured by a passive infrared sensor that detected changes in animal thermal radiation due to movement^31^. The sensor detected a change in the intensity of infrared energy radiated from an animal (The Chronobiology Kit, Stanford Software Systems, Stanford, CA). The amount of movement was recorded every minute with computer software Analysis98 (Stanford Software Systems, Santa Crus, CA).

### Open field test

A mouse was placed in an acrylic box (45 × 45 × 45 cm) for 70 min. The inside of the box was covered by rough-surfaced polypropylene sheets. The light intensity in the box was adjusted to 20 lx. The movement of each mouse was monitored through a CCD camera and was tracked using a software package (LimeLight, Actimetrics, USA). We considered the total distance traveled and the number of total crossings (defined by crossings of the lines made by the division of the chamber into 7.5 cm × 7.5 cm squares) as measures of locomotor activity. Moreover, we considered the percentage of time spent in the central area (15 cm × 15 cm square) as a measure of anxiety-like behavior.

### Y maze test

The details of the Y maze test have been described in our previous studies^27,32^. Briefly, a mouse was placed in an apparatus consisting of three arms (10 cm-wide, 45 cm-length, and 35 cm-high-walls) for 8 min. The light intensity in the apparatus was adjusted to 20 lx. The number of entries into an arm was as a measure of locomotor activity. The percentage of spontaneous alternation was used as a measure of working memory.

### 3-choice serial reaction time task (3-CSRTT)

Mice were trained to perform the 3-CSRTT as described previously^33^. We purchased aluminum operant chambers from Med Associates Inc. (St. Albans, VT, USA). The main sequence of the 3-CSRTT is briefly described below. When a mouse entered the food magazine, a 5-s inter-trial interval (ITI) began. After the ITI, one of the three hole lights was turned on (stimulus duration (SD) in experimental sessions: 1 s (SD1)) with a pseudo-random order. Nose poking before turning on a hole light was recorded as a “premature response,” which is a measure of impulsive action. Nose poking into the lit hole was recorded as a correct response and resulted in delivery of a palatable food pellet (20 mg, dustless precision pellets, Bio-Serv, Frenchtown, NJ, USA). Nose poking into an unlit hole was recorded as an incorrect response. When the animal did not nose poke into any holes, we recorded it as an omission. A 5-s time-out period started after premature responses, incorrect responses, and omissions. We also recorded the premature response latency (the time between the ITI onset and a nose poke into a unlit hole), the correct response latency (the time between stimulus onset and a nose poke into the lit hole), and reward latency (the time between reward delivery and a nose poke into the food magazine).

**Figure 6:**
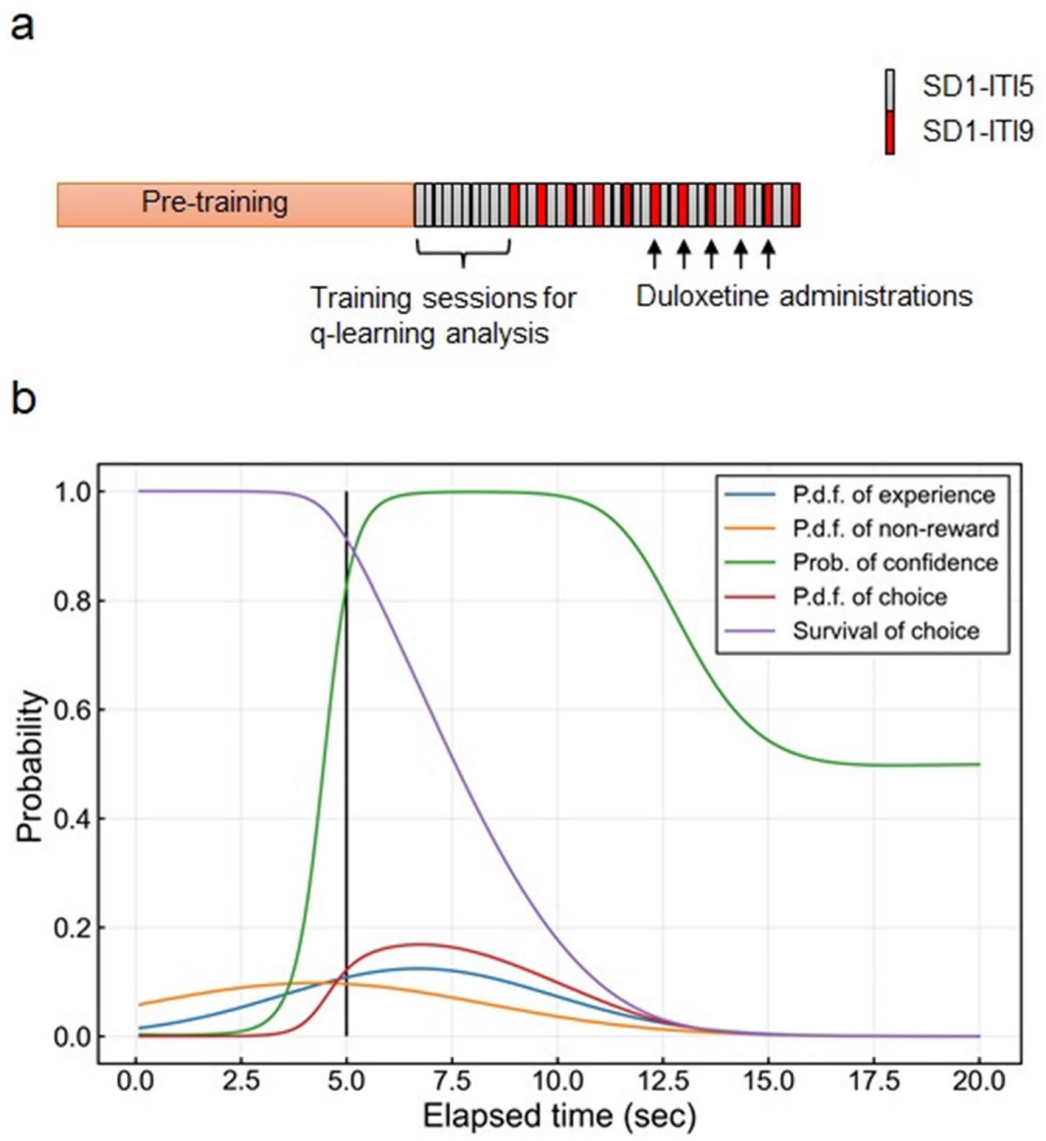
Sessions used for q-learning analysis and duloxetine administrations and illustration of the functions used in the q-learning model in the 3-choice serial reaction time task. (**a**) Training sessions after a pre-training period were used for q-learning analysis to assess the learning process. A gray or red box indicates one session. Duloxetine was administered after five SD1-ITI9 were completed. SD1-ITI5 stands for stimulus duration of one second and inter trial interval (ITI) of 5 s. SD1-ITI9 stands for stimulus duration of one second and inter trial interval (ITI) of 9 s. (**b**) X-axis represents elapsed time (sec) from the starting time of trials. The vertical black line at 5 s indicates the timing of the light stimulus. Each line represents the following: Probability density function (p.d.f.) of experience (blue); the p.d.f. of experience distribution. P.d.f. of non-reward (orange); the p.d.f. of non-reward distribution. Prob. of confidence (green); the probability of confidence at each elapsed time. P.d.f. of choice (red); the p.d.f. of choice distribution. Survival of choice (purple): the survival function of the choice distribution.

Session data in the 3-CSRTT were used for two purposes (Fig. 6a). Training sessions after a pre-training period were used for q-learning analysis to assess learning ability. The pre-training sessions included several types of training and mice usually experienced each step for only a few sessions. After five SD1-ITI9 sessions were completed, duloxetine administration was started as described later.

### Assessment of learning ability with a q-learning model with 3-CSRTT training sessions

We focused on ITI with 5-s (ITI5) training session data from the first session after the pre-training process to the tenth session (Fig. 6a). In these sessions, our preliminary analysis showed no clear difference in proportions of result categories (correct, premature, incorrect, or omission) between genotypes (see Supplementary Fig. 2). However, an impulsive action could be related to previous trial behaviors, and detailed analysis with a q-learning model could reveal differences between wild type and D5KO mice in terms of impulsivity. Premature response latency was recorded for these sessions and combined with correct and incorrect latencies. We could reconstruct the time between stimulus onset and a nose poke into the hole regardless of trial results.

The q-learning model in this study attempts to capture the mechanisms of premature behavior, which represents an impulsive action. To model the memory state in the mouse brain, we assumed two types of time-dependent probability distribution functions (p.d.f.) to represent “experience” and “non-reward” memory states. The experience distribution represents the memory of all trials that mice have completed, and a non-reward distribution represents the memory of trials with premature results. Combining these two mechanisms, mice decide when to nose poke into a hole. Parameters of both distributions are updated based on the q-learning process. Since incorrect results were thought to be caused by different mechanisms and the number of incorrect results was small, incorrect results were treated the same as correct ones in this analysis.

We firstly define random variables for “experience” and “non-reward” distributions, which follow a normal distribution. With q-learning theory, these two random variables were updated based on a result type and elapsed time from a start timing of the previous trial. Rates of updating each parameter are controlled by learning rates of the experience distribution, ${\alpha }_{X}$, and the non-reward distribution, ${\alpha }_{Y}$. If these learning rates are lower for D5KO mice than wild-type mice, we state that D5KO mice have a deficit in learning ability.

Experience and non-reward distributions are used to calculate the probability of confidence with a softmax function. An inverse temperature, $\beta$, in the softmax function controls the degree of confidence by weighting experience and non-reward distribution. If the inverse temperature is higher for D5KO mice than wild-type mice, D5KO mice have strong confidence in their own choices. This probability of confidence is multiplied by the experience distribution and scaled to one, yielding the probability density function of choice representing the time of the decision to nose poke into a hole. This function can be converted to be a survival function, which is used for simulation purposes. These probabilities and distributions are illustrated in Fig. 6b.

Detailed explanations of model derivation, parameter specification, estimation procedures, and simulation procedures are in Supplementary Information.

### The effects of acute duloxetine injection on impulsive action in mice

To determine whether dopamine D_5_ receptors play an important role in the enhancement of impulse control, we administered duloxetine (0, 0.3, 1.0, and 3.0 mg/kg) intraperitoneally to D5KO mice and their wildtype littermates 30 min before the 3-choice serial reaction time task session. We did not use higher doses of duloxetine (> 3.0 mg/kg) because higher doses induced sedation in our preliminary study. Drug treatments were carried out using a Latin square design and were administered with at least a 2-day interval between injections. During the testing phase of this study, the duration of the ITI was prolonged to 9 s (ITI9) because the mice made only a few (< 10) premature responses during the task using a 5-s interval (ITI5). Each testing session with ITI9 was conducted for 70 min or until 100 trials were completed, whichever came first, while sessions with ITI5 were conducted for 60 min or until 100 trials were completed, whichever came first. When the mice experienced 10 ITI5 sessions, they were habituated to ITI9 sessions 6 times with 2-day intervals.

The following behavioral measures in the 3-CSRTT were analyzed:

- Percentage of premature responses: [premature responses/(premature + correct + incorrect responses)] × 100, a measure of impulsive action
- Accuracy (percentage of correct responses): [correct responses/(correct + incorrect responses)] × 100, a measure of attentional function
- Percentage of omissions [(number of omissions/total initiated trials) × 100], a measure of attentional function and motivation for the task
- Correct response latency (s), a measure of attentional function, motivation for the task, and motor function
- Reward latency (s), a measure of motivation for reward and motor function

### Statistical analysis

For the measurement of locomotor activity in the home cage and in the open field test, we used a three-factor mixed analysis of variance (ANOVA) with time as a within-subjects factor and genotype and sex as between-subjects factors. For the effect of genotype in the Y maze test, we used a two-factor mixed ANOVA with genotype and sex as between-subjects factors. For the 3-CSRTT, each measure was analyzed separately by a three-factor mixed ANOVA with drug as a within-subjects factor and genotype and sex as between-subjects factors except for the assessment of learning ability. If Mauchly's sphericity test was significant, a Greenhouse–Geisser correction was used. Multiple comparisons with Bonferroni's correction were also conducted in cases where ANOVA revealed a significant main effect. All results except for the assessment of learning ability are presented as mean ± standard error of the mean (S.E.M.). The results were considered statistically significant when *p* < 0.05. SPSS (version 23.0) and GraphPad Prism (version 8.4.2) were used for statistical analyses.

## Supplementary Information

Supplementary Information.

## Data Availability

The datasets of this study are available from the corresponding author on reasonable request.

## Code availability

Codes used for model fitting and plotting of q-learning assessments of the 3-choice serial reaction time task is available on a GitHub repository at https://github.com/Neuropharmacol/3csrtt_q_learning.
