# Supplementary material for: Behavioral characteristics of dopamine D5 receptor knockout mice
Source: Sci Rep. 2022 Apr 10;12:6014. doi: 10.1038/s41598-022-10013-5 (PMC8995362; doi:10.1038/s41598-022-10013-5)
Supplement: Supplementary file 1 — Supplementary Information. [file 41598_2022_10013_MOESM1_ESM.docx]

Supplementary Information for:

**Behavioral characteristics of dopamine D5 receptor knockout mice**

Hitomi Sasamori^1†^, Toshiaki Asakura^2†^, Chiaki Sugiura^1†^, Youcef Bouchekioua^1^, Naoya Nishitani^1,3^, Masaaki Sato^1^, Takayuki Yoshida^1,4^, Miwako Yamasaki^5^, Akira Terao^6^, Masahiko Watanabe^5^, Yu Ohmura^1*^, and Mitsuhiro Yoshioka^1^^

^1^ Department of Neuropharmacology, Hokkaido University Faculty of Medicine, Sapporo, Japan

^2^ Hokkaido University School of Medicine, Sapporo, Japan

^3^ Laboratory of Molecular Pharmacology, Institute of Medical, Pharmaceutical and Health Sciences, Kanazawa University, Kanazawa, Japan

^4^ Department of Neurophysiology, Graduate School of Biomedical and Health Sciences, Hiroshima University, Hiroshima, Japan

^5^ Department of Anatomy, Hokkaido University Faculty of Medicine, Sapporo, Japan

^6^ Department of Biology, School of Biological Sciences, Tokai University, Sapporo, Japan

**† These authors contributed equally to this work.**

**^ This author deceased.**

* Correspondence:
Yu Ohmura

[yohmura@med.hokudai.ac.jp](mailto:yohmura@med.hokudai.ac.jp)

**This PDF file includes**:

Q-learning model for assessment of learning ability with 3-CSRTT training sessions

Model derivation

Decision process

Parameter specification

Estimation procedures

Simulation procedures

References

Tables S1 and S6.

Figures S1 and S2.

**Q-learning model for assessment of learning ability with 3-CSRTT training sessions**

Model derivation

To determine learning deficits or biases in D5KO mice, we modeled the mechanism of premature responses. We assume two types of time-dependent probability density functions (p.d.f.) to represent “experience” and “non-reward” memory states. The experience and non-reward distributions represent the memory of all trials and trials with premature results, respectively.

Let random variables for the experience and non-reward distributions at trial *n* be *X*_n_ and *Y*_n_, following normal distributions with mean $\mu_{X,n}$ and $\mu_{Y,n}$, and with variance $\sigma_{X,n}^{2}$ and $\sigma_{Y,n}^{2}$, respectively. Updating procedures for q-learning are performed depending on results of the trials. For the experience distribution, parameters are updated as follows. If trial results are correct or incorrect, parameters are updated as,

$$\begin{aligned} \boldsymbol{X}_{\boldsymbol{n+1}}\boldsymbol{=}\left( \boldsymbol{1-}\boldsymbol{\alpha}_{\boldsymbol{X}}\boldsymbol{-}\boldsymbol{\alpha}_{\boldsymbol{M}} \right)\boldsymbol{X}_{\boldsymbol{n}}\boldsymbol{+}\boldsymbol{\alpha}_{\boldsymbol{X}}\boldsymbol{R}_{\boldsymbol{n}}\boldsymbol{+}\boldsymbol{\alpha}_{\boldsymbol{M}}\boldsymbol{M.}\#\left( 1.1 \right) \end{aligned}$$

If premature,

$$\begin{aligned} \boldsymbol{X}_{\boldsymbol{n+1}}\boldsymbol{=}\left( \boldsymbol{1-}\boldsymbol{\alpha}_{\boldsymbol{X}} \right)\boldsymbol{X}_{\boldsymbol{n}}\boldsymbol{+}\boldsymbol{\alpha}_{\boldsymbol{X}}\boldsymbol{R}_{\boldsymbol{n}}\boldsymbol{.}\#\left( 1.2 \right) \end{aligned}$$

If omission, we do not update an experience distribution. Then,

$$\begin{aligned} \boldsymbol{X}_{\boldsymbol{n+1}}\boldsymbol{=}\boldsymbol{X}_{\boldsymbol{n}}\mathbf{.}\#\left( 1.3 \right) \end{aligned}$$

In the equations, *R*_n_ represents the elapsed time perception perceived by mice at the *n-*th trial, following a normal distribution with mean *t*_n_ and variance $\sigma_{r}^{2}$, where *t*_n_ is the actual nose-poking timing of mice at *n*-th trial. Even though a measured value is fixed to be one value, a mouse perceives its own trial timing as a ranged value around the actual trial timing. *M* is an adjustment random variable caused by a hole light in the experiment and follows a normal distribution with mean$t_{M}$ and $\sigma_{M}^{2}$, where $t_{M}$ is the actual timing of light flashing and is set to be 5 sec. The parameters $\alpha_{X}$ and $\alpha_{M}$ represent a learning rate for a random variable of the experience distribution, *R*_n_, and for adjustment term, *M*, respectively. A serial number for *n*-th trial is assigned to the entire experiment for trials of each mouse.

Since these random variables follow normal distributions, the reproductive property of the normal distribution holds. Therefore, we can rewrite the equations as follows. If trial results are correct or incorrect,

$$\begin{aligned} \boldsymbol{\mu}_{\boldsymbol{X,n+1}}\boldsymbol{=}\left( \boldsymbol{1-}\boldsymbol{\alpha}_{\boldsymbol{X}}\boldsymbol{-}\boldsymbol{\alpha}_{\boldsymbol{M}} \right)\boldsymbol{\mu}_{\boldsymbol{X,n}}\boldsymbol{+}\boldsymbol{\alpha}_{\boldsymbol{X}}\boldsymbol{t}_{\boldsymbol{n}}\boldsymbol{+}\boldsymbol{\alpha}_{\boldsymbol{M}}\boldsymbol{t}_{\boldsymbol{M}}\boldsymbol{,}\#\left( 2.1 \right) \end{aligned}$$

$$\begin{aligned} \boldsymbol{\sigma}_{\boldsymbol{X,n+1}}^{\boldsymbol{2}}\boldsymbol{=}\left( \boldsymbol{1-}\boldsymbol{\alpha}_{\boldsymbol{X}}\boldsymbol{-}\boldsymbol{\alpha}_{\boldsymbol{M}} \right)^{\boldsymbol{2}}\boldsymbol{\sigma}_{\boldsymbol{X,n}}^{\boldsymbol{2}}\boldsymbol{+}\boldsymbol{\alpha}_{\boldsymbol{X}}^{\boldsymbol{2}}\boldsymbol{\sigma}_{\boldsymbol{r}}^{\boldsymbol{2}}\boldsymbol{+}\boldsymbol{\alpha}_{\boldsymbol{M}}^{\boldsymbol{2}}\boldsymbol{\sigma}_{\boldsymbol{M}}^{\boldsymbol{2}}\boldsymbol{.}\#\left( 2.2 \right) \end{aligned}$$

If premature,

$$\begin{aligned} \boldsymbol{\mu}_{\boldsymbol{X,n+1}}\boldsymbol{=}\left( \boldsymbol{1-}\boldsymbol{\alpha}_{\boldsymbol{X}} \right)\boldsymbol{\mu}_{\boldsymbol{X,n}}\boldsymbol{+}\boldsymbol{\alpha}_{\boldsymbol{X}}\boldsymbol{t}_{\boldsymbol{n}}\boldsymbol{,}\#\left( 2.3 \right) \end{aligned}$$

$$\begin{aligned} \boldsymbol{\sigma}_{\boldsymbol{X,n+1}}^{\boldsymbol{2}}\boldsymbol{=}\left( \boldsymbol{1-}\boldsymbol{\alpha}_{\boldsymbol{X}} \right)^{\boldsymbol{2}}\boldsymbol{\sigma}_{\boldsymbol{X,n}}^{\boldsymbol{2}}\boldsymbol{+}\boldsymbol{\alpha}_{\boldsymbol{X}}^{\boldsymbol{2}}\boldsymbol{\sigma}_{\boldsymbol{r}}^{\boldsymbol{2}}\boldsymbol{.} \#\left( 2.4 \right) \end{aligned}$$

If omission, we do not update an experience distribution. Then,

$$\begin{aligned} \boldsymbol{\mu}_{\boldsymbol{X,n+1}}\boldsymbol{=} \boldsymbol{\mu}_{\boldsymbol{X,n}}\boldsymbol{,}\#\left( 2.5 \right) \end{aligned}$$

$$\begin{aligned} \boldsymbol{\sigma}_{\boldsymbol{X,n+1}}^{\boldsymbol{2}}\boldsymbol{=}\boldsymbol{\sigma}_{\boldsymbol{X,n}}^{\boldsymbol{2}}\boldsymbol{.}\#\left( 2.6 \right) \end{aligned}$$

Similarly, a non-reward distribution is defined, but there are two different points. Updates are done only if premature response occurred and there is no adjustment random variable since a mouse does not see a hole light when a result is premature. If premature response occurred, the q-learning process for a non-reward distribution is defined as

$$\begin{aligned} \boldsymbol{Y}_{\boldsymbol{n+1}}\boldsymbol{=}\left( \boldsymbol{1-}\boldsymbol{\alpha}_{\boldsymbol{Y}} \right)\boldsymbol{Y}_{\boldsymbol{n}}\boldsymbol{+}\boldsymbol{\alpha}_{\boldsymbol{Y}}\boldsymbol{R}_{\boldsymbol{n}}\boldsymbol{.}\#\left( 3.1 \right) \end{aligned}$$

Otherwise,

$$\begin{aligned} \boldsymbol{Y}_{\boldsymbol{n+1}}\boldsymbol{=}\boldsymbol{Y}_{\boldsymbol{n}}\boldsymbol{,}\#\left( 3.2 \right) \end{aligned}$$

where $\alpha_{Y}$ represents a learning rate for random variables of a non-reward distribution. Since *Y*_n_ and *R*_n_ follow normal distributions, we can again rewrite theses equations. If premature,

$$\begin{aligned} \boldsymbol{\mu}_{\boldsymbol{Y,n+1}}\boldsymbol{=}\left( \boldsymbol{1-}\boldsymbol{\alpha}_{\boldsymbol{Y}} \right)\boldsymbol{\mu}_{\boldsymbol{Y,n}}\boldsymbol{+}\boldsymbol{\alpha}_{\boldsymbol{Y}}\boldsymbol{t}_{\boldsymbol{n}}\boldsymbol{,}\#\left( 4.1 \right) \end{aligned}$$

$\begin{aligned} \boldsymbol{\sigma}_{\boldsymbol{Y,n+1}}^{\boldsymbol{2}}\boldsymbol{=}\left( \boldsymbol{1-}\boldsymbol{\alpha}_{\boldsymbol{Y}} \right)^{\boldsymbol{2}}\boldsymbol{\sigma}_{\boldsymbol{Y,n}}^{\boldsymbol{2}}\boldsymbol{+}\boldsymbol{\alpha}_{\boldsymbol{Y}}^{\boldsymbol{2}}\boldsymbol{\sigma}_{\boldsymbol{r}}^{\boldsymbol{2}}\boldsymbol{.}\#\left( 4.2 \right) \end{aligned}$Otherwise, the parameters are not updated.

**Decision process**

Experience and non-reward distributions are used to model the decision process of when to start a trial. We assume that the decision is made based on the experiences and confidence in success. The probability of confidence in success at time *t* at *n*-th trial is derived with a softmax function as

$$\begin{aligned} \boldsymbol{P}_{\boldsymbol{conf,n}}\left( \boldsymbol{t} \right)\boldsymbol{=}\frac{\boldsymbol{e}^{\boldsymbol{\beta P}_{\boldsymbol{X}}\left( \boldsymbol{t} \right)}}{\boldsymbol{e}^{\boldsymbol{\beta P}_{\boldsymbol{X}}\left( \boldsymbol{t} \right)}\boldsymbol{+}\boldsymbol{e}^{\boldsymbol{\beta P}_{\boldsymbol{Y}}\left( \boldsymbol{t} \right)}}\boldsymbol{,}\#\left( 5 \right) \end{aligned}$$

where $P_{X}\left( t \right)=P(t ;\mu_{X,n},\sigma_{X,n}^{2}) , P_{Y}\left( t \right)=P(t ;\mu_{Y,n},\sigma_{Y,n}^{2})$, and β is an inverse temperature. As learning proceeds, this probability of confidence becomes below 0.5 before a hole light flashing, above 0.5 just after a hole light flashing, and 0.5 long after light flashing. This movement of the probability of confidence is similar to our thinking. When a trial starts, we do not concentrate on whether it is a good time to start or not because we know that the probability of success is too small. However, if flash timing is coming, we concentrate on whether this start timing results in success or not. A long time after light flashing, again, we do not concentrate on whether this start timing results in success or not.

This probability of confidence is used for elementwise multiplication over time to the experience distribution at *n*-th trial, and the obtained distribution is scaled to one on the positive interval, yielding the p.d.f. of the choice distribution at *n*-th trial, $P_{d,n}(t)$. The survival function of this choice distribution was used for simulation. Premature responses, along with correct and incorrect latencies, were used to calculate the time between stimulus onset and nose poke into the hole regardless of trial results.

Figure 6b illustrates the distributions and the probabilities defined above with example parameters. These distributions were updated according to each trial session results. The p.d.f. of experience (blue) and non-reward distribution (orange) were normal distributions. The probability of confidence was not a distribution but a set of probabilities over time and multiplied to the p.d.f. of experience distribution, yielding the choice distribution. The survival function of the choice distribution was used for a simulation purpose.

**Parameter specification**

Our aim was to assess differences in various aspects of learning ability between wildtype and D5KO mouse. Then, key parameters for the learning process were divided into baseline, sex, and genetic effects. In the equations (2.1) - (2.4), (4.1), (4.2), and (5), $\alpha_{X}$*,* $\alpha_{Y},$ and β were divided as

$$\begin{aligned} \boldsymbol{\alpha}_{\boldsymbol{X}}\boldsymbol{=}\boldsymbol{\alpha}_{\boldsymbol{X,0}}\boldsymbol{+}\boldsymbol{\alpha}_{\boldsymbol{X,male}}\boldsymbol{+}\boldsymbol{\alpha}_{\boldsymbol{X,D}\boldsymbol{5}\boldsymbol{KO}}\boldsymbol{,}\#\left( 6.1 \right) \end{aligned}$$

$$\begin{aligned} \boldsymbol{\alpha}_{\boldsymbol{Y}}\boldsymbol{=}\boldsymbol{\alpha}_{\boldsymbol{Y,0}}\boldsymbol{+}\boldsymbol{\alpha}_{\boldsymbol{Y,male}}\boldsymbol{+}\boldsymbol{\alpha}_{\boldsymbol{Y,D}\boldsymbol{5}\boldsymbol{KO}}\boldsymbol{,}\#\left( 6.2 \right) \end{aligned}$$

and

$$\begin{aligned} \boldsymbol{\beta}\boldsymbol{=}\boldsymbol{\beta}_{\boldsymbol{0}}\boldsymbol{+}\boldsymbol{\beta}_{\boldsymbol{male}}\boldsymbol{+}\boldsymbol{\beta}_{\boldsymbol{D}\boldsymbol{5}\boldsymbol{KO}}\boldsymbol{.}\#\left( 6.3 \right) \end{aligned}$$

Table S4 shows the list of q-learning analysis parameters to be estimated in this analysis. A total of 14 parameters were estimated in this study. In addition to learning rates, inverse temperatures, and q-learning related parameters, initial values of variances for the experience, $\sigma_{X,0}^{2}$, and non-reward distribution, $\sigma_{Y,0}^{2}$, were also estimated. In total, 14 parameters were estimated in this analysis. Here, we assumed these variances were the same for every mouse. For the initial values of the mean of the experience, $\mu_{X,0}$, and non-reward distribution, $\mu_{Y,0}$, we used the mean values of the first training session to reflect the different achievement levels after pre-training sessions.

**Estimation procedures**

Q-learning related parameters were estimated by the maximum likelihood method. Let $\boldsymbol{\theta}$ be parameters to be estimated and the likelihood function for this model is given by,

$$\begin{aligned} \boldsymbol{L}\left( \boldsymbol{\theta} \right)\boldsymbol{=}\prod_{\boldsymbol{n=1}}^{\boldsymbol{N}} \boldsymbol{P}_{\boldsymbol{d,n-1}}\left( \boldsymbol{t}_{\boldsymbol{n}}\boldsymbol{;\theta} \right)\boldsymbol{,}\#\left( 7 \right) \end{aligned}$$

where *N* is the total number of trials, and $P_{d,n-1}$ is the p.d.f. of the choice distribution at (*n-1)*-th trial.

For this analysis, 46 mice were used, including 14 wildtype male mice, 14 wildtype female mice, 11 D5KO male mice, and 10 D5KO female mice.

Estimation was performed by ensemble sampling implemented in emcee, one of the Python packages^1^. We ran 19000 steps with 300 walkers and discarded the first 3000 samples and thinned every 200. We also estimated listed parameters individually to show how this model can capture the learning process. Since this model is not affected by omission results, we omitted all omission trials when estimating.

**Simulation procedures**

Our model has a lot of parameters, and it is difficult to interpretate estimates of each parameter. Therefore, simulations from estimated parameters were performed to understand the contributions of each parameter to the behavior. Let *N*_s_ be the pre-defined number of trials. The simulations were performed as follows.

**Step 1.** Set n = 1.

**Step 2**. Generate *U*, uniform random variable with the interval [0,1]

**Step 3.** Choose $t_{n}$ at which the survival function of the choice distribution at (*n-1)*-th trial takes U.

**Step 4.** Update parameters using the equations (2.1) – (2.4), (4.1) and (4.2).

**Step 5.** If *n* becomes *N*_s_, finish. Otherwise, set n = n+1 and go to Step 2.

Using this procedure, we can simulate the q-learning process. Since the average number of each training trial session was 87, we simulated 900 steps and divided these steps into 10 parts to correspond to sessions of real data. These procedures were performed 100 times, and averaged values were regarded as simulated results. The analysis of assessment of learning ability was done with Python 3.8.5.

**References**

1. Foreman-Mackey, D., Hogg, D. W., Lang, D., & Goodman, J. (2013). emcee: the MCMC hammer. *Publications of the Astronomical Society of the Pacific*, *125*(925), 306.

**Supplementary Table S1.** Results of three-factor ANOVA on the number of spontaneous movements per 2 hours in home cages. Related to Figure 2a.

| Parameter | Factor | *F* | *DF* | *P* value |
| --- | --- | --- | --- | --- |
| Number of spontaneous movements per 2 hours | Time^1^ | 115.300 | 5.39, 285.4 | <0.001** |
|  | Sex | 0.002 | 1, 53 | 0.965 |
|  | Genotype | 0.665 | 1, 53 | 0.419 |
|  | Time × Sex | 1.924 | 11, 583 | 0.034* |
|  | Time × Genotype | 3.336 | 11, 583 | <0.001 |
|  | Sex × Genotype | 2.000 | 1, 53 | 0.163 |
|  | Time × Sex × Genotype | 1.475 | 11, 583 | 0.136 |

**P*<0.05; ***P*<0.01.

^1^ Greenhouse-Geisser correction was applied.

**Supplementary Table S2.** Results of two-factor ANOVA on parameters of the open field test. Related to Figures 2b, 2c, and 2d.

| Parameter | Factor | *F* | *DF* | *P* value |
| --- | --- | --- | --- | --- |
| Distance traveled  over the testing period | Time^1^ | 119.100 | 3.450, 169.0 | <0.001** |
|  | Sex | 0.287 | 1, 49 | 0.595 |
|  | Genotype | 0.820 | 1, 49 | 0.370 |
|  | Time × Sex | 1.854 | 6, 294 | 0.089 |
|  | Time × Genotype | 0.422 | 6, 294 | 0.865 |
|  | Sex × Genotype | 2.114 | 1, 49 | 0.152 |
|  | Time × Sex × Genotype | 0.847 | 6, 294 | 0.535 |
| Number of crossings | Time | 93.960 | 6, 294 | <0.001** |
|  | Sex | 0.258 | 1, 49 | 0.614 |
|  | Genotype | 0.072 | 1, 49 | 0.790 |
|  | Time × Sex | 1.320 | 6, 294 | 0.248 |
|  | Time × Genotype | 0.469 | 6, 294 | 0.831 |
|  | Sex × Genotype | 2.858 | 1, 49 | 0.097 |
|  | Time × Sex × Genotype | 0.958 | 6, 294 | 0.454 |
| Time spent  in the central area (%) | Time^1^ | 6.858 | 2.989, 146.5 | <0.001** |
|  | Sex | 3.252 | 1, 49 | 0.078 |
|  | Genotype | 0.979 | 1, 49 | 0.327 |
|  | Time × Sex | 1.686 | 6, 294 | 0.124 |
|  | Time × Genotype | 0.927 | 6, 294 | 0.476 |
|  | Sex × Genotype | 0.065 | 1, 49 | 0.800 |
|  | Time × Sex × Genotype | 1.723 | 6, 294 | 0.115 |

**P*<0.05; ***P*<0.01.

^1^ Greenhouse-Geisser correction was applied.

**Supplementary Table S3.** Results of two-factor ANOVA on parameters of the Y maze test. Related to Figure 3.

| Parameter | Factor | *F* | *DF* | *P* value |
| --- | --- | --- | --- | --- |
| Spontaneous alteration (%) | Sex | 0.466 | 1, 52 | 0.498 |
|  | Genotype | 0.886 | 1, 52 | 0.351 |
|  | Sex × Genotype | 3.904 | 1, 52 | 0.054 |
| Arm entries (counts) | Sex | 0.299 | 1, 52 | 0.587 |
|  | Genotype | 0.020 | 1, 52 | 0.888 |
|  | Sex × Genotype | 0.009 | 1, 52 | 0.927 |

**P*<0.05; ***P*<0.01.

**Supplementary Table S4. List of q-learning related parameters to be estimated.**

| **Symbols** | **Explanations** |
| --- | --- |
| $\alpha_{X,0},\alpha_{X,male}, \alpha_{X, D5KO}$ | Baseline, male, and D5KO effects of learning rates for the experience distribution. |
| $\alpha_{Y,0},\alpha_{Y,male}, \alpha_{Y, D5KO}$ | Baseline, male, and D5KO effects of learning rates for the non-reward distribution. |
| $\beta_{0},\beta_{male}, \beta_{D5KO}$ | Inverse temperature of baseline, male, and D5KO effects. |
| $\alpha_{M}$ | Learning rate for the adjustment term. |
| $\sigma_{M}^{2}$ | Variance of learning rate for the adjustment term. |
| $\sigma_{r}^{2}$ | Variance of the distribution of actual start timing. |
| $\sigma_{M}^{2}$ | Variance of the distribution of adjustment. |
| $\sigma_{X,0}^{2}$, $\sigma_{Y,0}^{2}$ | Initial values of variances for the experience and non-reward distribution, respectively. |

**Supplementary Table S5.** Proportions of premature responses for each session for trial and simulation data. ⁋

|  | Trial data | | | | Simulations | | | |
| --- | --- | --- | --- | --- | --- | --- | --- | --- |
| Session | D5KO / male | D5KO / female | WT / male | WT / female | D5KO / male | D5KO / female | WT / male | WT / female |
| 1 | 29.73 | 32.20 | 30.93 | 33.36 | 25.70 | 25.50 | 26.30 | 25.46 |
| 2 | 39.09 | 37.10 | 32.14 | 35.45 | 14.93 | 16.43 | 15.84 | 16.88 |
| 3 | 27.73 | 24.70 | 35.14 | 38.00 | 11.94 | 15.01 | 14.93 | 16.58 |
| 4 | 29.91 | 23.00 | 27.00 | 34.64 | 12.57 | 15.41 | 15.40 | 16.52 |
| 5 | 20.64 | 21.30 | 32.71 | 29.09 | 12.42 | 14.93 | 15.04 | 16.88 |
| 6 | 22.09 | 15.90 | 23.50 | 24.73 | 13.16 | 15.40 | 14.68 | 17.13 |
| 7 | 29.55 | 36.70 | 21.21 | 22.73 | 13.44 | 15.10 | 14.61 | 15.83 |
| 8 | 25.82 | 22.10 | 29.79 | 15.91 | 13.44 | 15.44 | 15.74 | 16.83 |
| 9 | 21.82 | 26.10 | 18.86 | 13.36 | 12.13 | 16.42 | 15.38 | 16.16 |
| 10 | 14.45 | 20.10 | 19.64 | 11.27 | 12.38 | 15.29 | 14.81 | 15.87 |

⁋ Proportions of premature responses for trial data were just simple division. Since the average number of each trial session was 87, we ran 900 steps for simulation and divided these steps into 10 groups and calculated the proportion of trials in which the elapsed time was less than 5.

**Supplementary Table S6.** Results of three-factor ANOVA on parameters of the duloxetine administration test. Related to Figure 5.

| Parameter | Factor | *F* | *DF* | *P* value |
| --- | --- | --- | --- | --- |
| Premature responses (%) | Drug | 14.063 | 2.51, 140.26 | <0.001** |
|  | Sex | 0.380 | 1, 56 | 0.540 |
|  | Genotype | 2.184 | 1, 56 | 0.145 |
|  | Drug × Sex | 2.332 | 2.51, 140.26 | 0.088 |
|  | Drug × Genotype | 0.335 | 2.51, 140.26 | 0.763 |
|  | Sex × Genotype | 0.040 | 1, 56 | 0.841 |
|  | Drug × Sex × Genotype | 0.008 | 2.51, 140.26 | 0.997 |
| Accuracy (%) | Drug | 3.934 | 2.56, 143.28 | 0.014* |
|  | Sex | 0.926 | 1, 56 | 0.340 |
|  | Genotype | 0.198 | 1, 56 | 0.658 |
|  | Drug × Sex | 0.475 | 2.56, 143.28 | 0.670 |
|  | Drug × Genotype | 0.181 | 2.56, 143.28 | 0.882 |
|  | Sex × Genotype | 1.986 | 1, 56 | 0.164 |
|  | Drug × Sex × Genotype | 0.944 | 2.56, 143.28 | 0.410 |
| Correct latency (s) | Drug | 10.661 | 2.40, 134.19 | <0.001** |
|  | Sex | 1.449 | 1, 56 | 0.234 |
|  | Genotype | 0.935 | 1, 56 | 0.338 |
|  | Drug × Sex | 0.984 | 2.40, 134.19 | 0.389 |
|  | Drug × Genotype | 0.454 | 2.40, 134.19 | 0.671 |
|  | Sex × Genotype | 3.556 | 1, 56 | 0.065 |
|  | Drug × Sex × Genotype | 1.153 | 2.40, 134.19 | 0.325 |
| Reward latency (s) | Drug | 6.735 | 3, 168 | <0.001** |
|  | Sex | 5.210 | 1, 56 | 0.003** |
|  | Genotype | 0.121 | 1, 56 | 0.729 |
|  | Drug × Sex | 2.157 | 3, 168 | 0.095 |
|  | Drug × Genotype | 1.093 | 3, 168 | 0.354 |
|  | Sex × Genotype | 0.268 | 1, 56 | 0.607 |
|  | Drug × Sex × Genotype | 0.408 | 3, 168 | 0.747 |
| Omission (%) | Drug | 1.193 | 3, 168 | 0.314 |
|  | Sex | 3.507 | 1, 56 | 0.066 |
|  | Genotype | 0.999 | 1, 56 | 0.322 |
|  | Drug × Sex | 1.702 | 3, 168 | 0.169 |
|  | Drug × Genotype | 0.228 | 3, 168 | 0.877 |
|  | Sex × Genotype | 1.095 | 1, 56 | 0.300 |
|  | Drug × Sex × Genotype | 2.657 | 3, 168 | 0.050 |

**P*<0.05; ***P*<0.01.

^1^ Greenhouse-Geisser correction was applied.


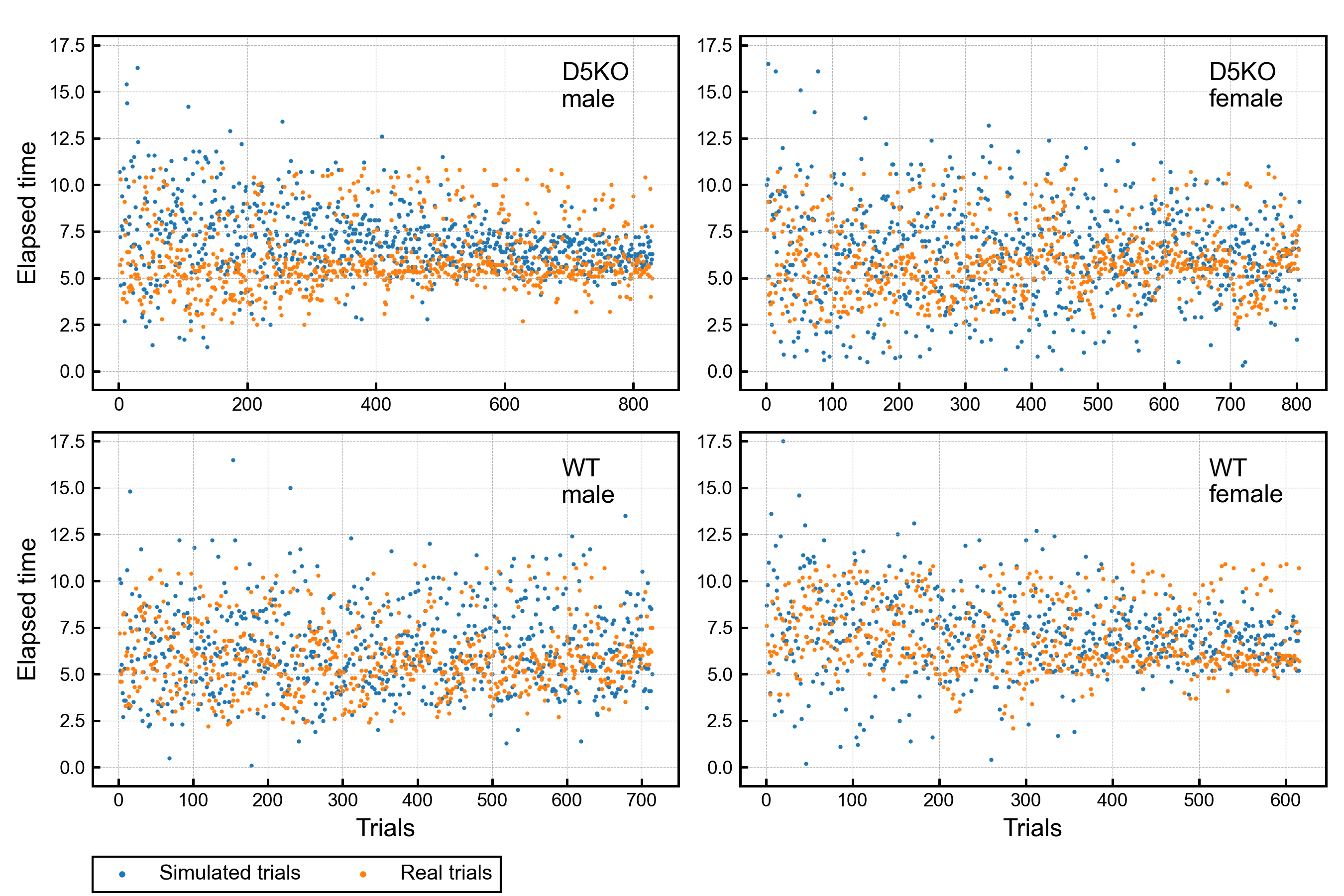


**Supplementary Figure 1. Scatter plot of simulated (blue) and real (orange) trials for each genomic and sex type.** X-axis represented n-th trial results from training sessions, and the y-axis represents elapsed time from stimulus onset to nose poke into any hole. Simulations were performed with individually estimated parameters with the same number of real trials for each mouse. It is noted that since omissions did not affect the q-learning process, we omitted omissions from visualization.


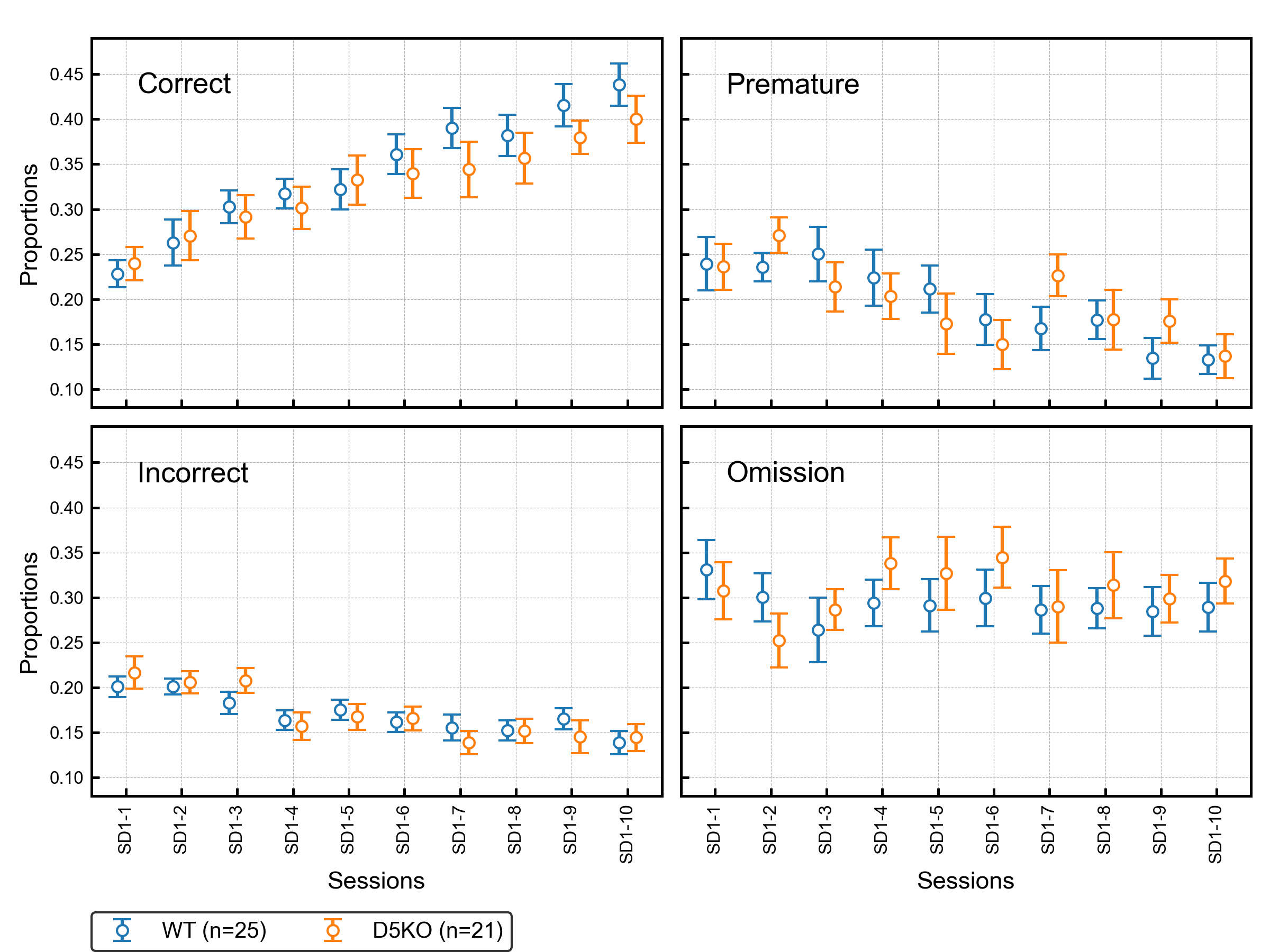


**Supplementary Figure 2. Proportions of each trial result category during training sessions stratified by genotype (wild and D5KO).** Result categories include correct, premature, incorrect, and omission. The circle represents the mean of each trial result category, and the error bar represents means ± S.E.M. SD1 stands for stimulus duration (SD) in experimental sessions: 1 s.
